# High-Speed Network DDoS Attack Detection: A Survey

**DOI:** 10.3390/s23156850

**Published:** 2023-08-01

**Authors:** Rana M. Abdul Haseeb-ur-rehman, Azana Hafizah Mohd Aman, Mohammad Kamrul Hasan, Khairul Akram Zainol Ariffin, Abdallah Namoun, Ali Tufail, Ki-Hyung Kim

**Affiliations:** 1 Center for Cyber Security, Faculty of Information Science and Technology, University Kebangsaan Malaysia (UKM), Bangi 43600, Malaysia; azana@ukm.edu.my (A.H.M.A.); mkhasan@ukm.edu.my (M.K.H.); k.akram@ukm.edu.my (K.A.Z.A.); 2 Faculty of Computer and Information Systems, Islamic University of Madinah, Madinah 42351, Saudi Arabia; a.namoun@iu.edu.sa; 3 School of Digital Science, Universiti Brunei Darussalam, Tungku Link, Gadong BE1410, Brunei; ali.tufail@ubd.edu.bn; 4 Department of Cyber Security, Ajou University, Suwon 16499, Republic of Korea

**Keywords:** denial of service, distributed denial of service, cyber–physical system, machine learning, high-speed network, intrusion detection system, express data path

## Abstract

Having a large number of device connections provides attackers with multiple ways to attack a network. This situation can lead to distributed denial-of-service (DDoS) attacks, which can cause fiscal harm and corrupt data. Thus, irregularity detection in traffic data is crucial in detecting malicious behavior in a network, which is essential for network security and the integrity of modern Cyber–Physical Systems (CPS). Nevertheless, studies have shown that current techniques are ineffective at detecting DDoS attacks on networks, especially in the case of high-speed networks (HSN), as detecting attacks on the latter is very complex due to their fast packet processing. This review aims to study and compare different approaches to detecting DDoS attacks, using machine learning (ML) techniques such as k-means, K-Nearest Neighbors (KNN), and Naive Bayes (NB) used in intrusion detection systems (IDSs) and flow-based IDSs, and expresses data paths for packet filtering for HSN performance. This review highlights the high-speed network accuracy evaluation factors, provides a detailed DDoS attack taxonomy, and classifies detection techniques. Moreover, the existing literature is inspected through a qualitative analysis, with respect to the factors extracted from the presented taxonomy of irregular traffic pattern detection. Different research directions are suggested to support researchers in identifying and designing the optimal solution by highlighting the issues and challenges of DDoS attacks on high-speed networks.

## 1. Introduction

With the increase in network traffic through the introduction of devices such as remote sensors, intelligent devices, self-drive Global Positioning System (GPS)-connected vehicles, 5G data transmission, smartphones, and cloud computing, the size of the internet is rapidly increasing [1]. There are approximately 4.66 billion internet users globally, which amounts to 59.5% of the global population. Similarly, approximately 53.6% of the global population are social media users, while smartphone users constitute 66.6%. Overall, the total population connected to the digital world was approximately 7.83 billion in 2021, with an anticipated annual growth of 316 million users. The expected internet user growth is alarming, especially when it comes to internet security and the integrity of Cyber–Physical Systems (CPS) [2]. Although the internet helps with different aspects of life and makes life more convenient, it creates many security risks. A typical example of these risks is malicious attacks such as DoS attacks, deception attacks, and reply attacks, all of which are types of cyber-attack. Their objectives and methods are different. DoS attacks aim to disrupt availability and deception attacks involve manipulation and trickery, whereas replay attacks focus on intercepting and reusing valid data to gain unauthorized access or manipulate systems. In addition, Denial-of-Service (DoS) attacks are related to breaches in user privacy and compromised security [3].

Generally, two forms of DoS attack are troubling, DoS and DDoS (DDoS). Typically, DDoS attacks occur through linked devices from numerous locations. The attack can cause unusual activity that interrupts the regular traffic of specific servers, services, and networks through data bombardment from nearby infrastructure. This unusual activity creates tremendous continuous service requests to the servers and networks, making it difficult to identify a trustworthy source. For example, in the Internet of things (IoT) environment, an attacker can quickly attack thousands of devices on a large scale [4,5,6,7]. For a practical CPS communication network, time delay is an important issue. A durable, adaptive DSC based on the dwell-time strategy and switching perspective was developed for a time-delayed switched nonlinear CPS under hybrid attacks on sensor measurements [8]. To investigate the stochastic characteristics of end-to-end network-induced time delay in a time-critical smart substation CPS context, the components of a smart substation CPS, such as data flow, communication network, and intelligent electronic devices (IEDs), are modelled [9]. In the case of time delay attacks (TDAs), which exploit communication channel weaknesses to cause potentially serious harm to a system, many of the approaches suggested for TDA detection have been evaluated exclusively offline and under strict assumptions of building a practical method for dealing with real-world problems [10]. DDoS attacks can be application layer attacks, protocol attacks, and volume-based attacks, and detecting them is more challenging on high-speed networks (HSNs). In HSNs, which consist of optical fiber networks with data rates of 100 Gbs, the context switching of network processing due to a DoS attack can reduce network speed due to a packet associated with a system call and a copy of the transition propagating across the network [11].

**Figure 1:**
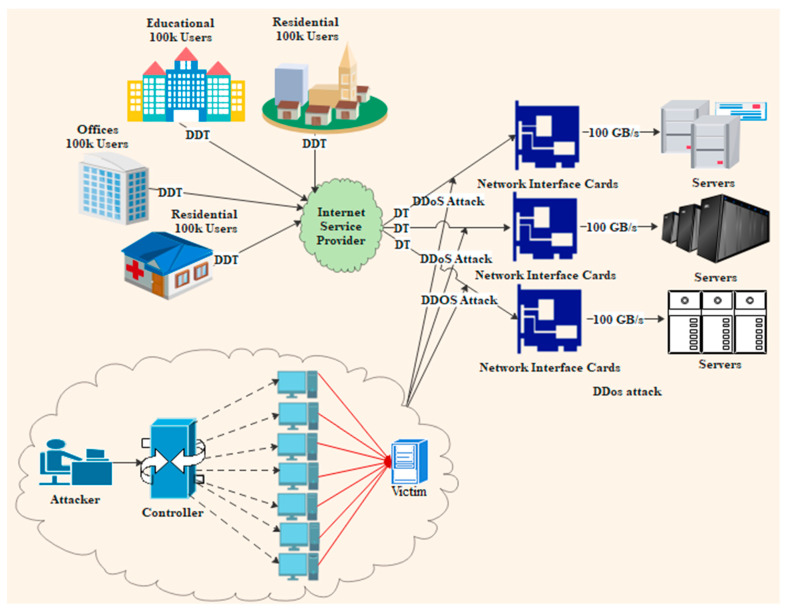
DDoS attack in a high-speed network scenario.

Since the speed of data processing on networks has grown, detecting DDoS attacks has become more complicated, raising security risks. Figure 1 illustrates a scenario of a DDoS attack occurring in a high-speed network. Additionally, researchers face enormous challenges in addressing DDoS attacks due to the network speed and different types of data entering the network [12]. Several DDoS attack detection techniques have been proposed, with two common types of detection, namely misuse detection and abnormal detection [13,14]. Both detection systems have limitations regarding the parameters selected for detecting network patterns. The advantage of misuse detection is that it provides a high accuracy; however, it requires complete information on the network. In contrast, prior knowledge of the network is not acquired in abnormal detection, but this approach does not provide the high accuracy offered by misuse detection [15].

In recent years, there have been several reviews in the literature of DoS attacks. For example, the authors of [16] presented the taxonomy of low-rate DoS attacks based on a three-layer modus operandi. The review included slow rate, service queue, and Quality of Service (QOS) attacks and described the various detection approaches against eight low-rate DoS attacks. However, the paper did not mention high-speed Network DDoS attacks. The authors of [17] presented cutting-edge defense techniques that help to prevent DDoS attacks and reduce the damage to user information. The review elaborates on the prevention techniques for IoT and Software-Defined Network devices. Ironically, DDoS attacks in a high-speed network scenario are not discussed. In [18], the authors described a defense mechanism against DDoS attacks, including the attack response, traffic classification, and attack detection, but not the network details. Motivated by the above observations, the aims of this review are to present:

- A comprehensive review of the types of DDoS attacks, detection, and prevention techniques;
- A survey of recent DDoS attacks on high-speed networks;
- An organized taxonomy of irregular traffic detection patterns in high-speed networks;
- A comprehensive investigation of the conventional weaknesses and strengths of DDoS detection techniques.

The paper’s road map is as follows; Section 2 elaborates on the research motive and background of high-speed data networks and illustrates the taxonomy of high-speed networks. The third section presents DDoS attack detection and prevention. The fourth section discusses the current state-of-the-art of high-speed network and the limitation of a DDoS attack. The fifth section explains the issues and challenges in high-speed data monitoring and analyzing networks for DDoS attack detection. The last section elaborates on the paper’s conclusion and future direction. The acronyms used in this paper are noted at the end of the paper.

## 2. Background of High-Speed Network

The development of network speed can be traced back to the 1960s, when the first computer networks were created. These networks allowed numerous computers to share resources, such as printers and data. Ethernet technology was invented in the 1980s, allowing computers to communicate at speeds of up to 10 Mbps [19]. This was followed in the 1990s by the creation of Fast Ethernet, which boosted this speed to 100 Mbps [20]. Gigabit Ethernet was created in the early 2000s, allowing for up to 1 Gbps rates. This was followed by 10 Gigabit Ethernet, which offers up to 10 Gbps rates [21]. Fast data transfer rates with a low latency, high bandwidth, and reliable and consistent performance are called high-speed networks (HSN). HSN technologies are being developed to enable even quicker and more efficient communication between devices. Among these technologies are 40 Gigabit Ethernet, 100 Gigabit Ethernet, and InfiniBand [22]. A Cisco Report predicted that the internet protocol may cross 4.3 zettabytes in 2023, which is 1879 exabytes higher than 2018 [3]. The variety of devices connected to the internet leads to massive data transfer on the network with a high velocity, which fulfils the critical demand of big data [18]. Smart devices face many security threats due to their internet connectivity and the various applications running on high-speed networks [23]. A high-speed communication network has a high bandwidth and low-latency communication. A data stream analysis is necessary for high-speed real-time data from many resources connected to the network [24]. According to [25], packet speed categories can be classified as shown in Table 1, while Table 2 shows the traffic types with their characteristics.

The vast amount of heterogeneous data gathered from network devices poses challenges in monitoring and detecting irregularities in the network [26]. The current network traffic supports IoT infrastructure with numerous devices, and big data stored in the cloud [27]. Mobile cloud is an emerging technology involved in different domains to reduce the mobile cloud (MC) limitations using cloud services due to connected wireless media [28]. Due to minimal storage resources, the data gathered from various smart devices is stored and processed in the cloud [29]. Cloud computing provides information technology services at various locations. For example, Mobile Cloud Computing (MCC) offers customer service on board [30]. Mobile health applications enable access to monitoring health activity electronically with the help of cloud services [31,32]. Nowadays, cloud service providers, such as Amazon, Google Cloud, Microsoft, IBM, Oracle, and many others, adopt big data technologies. The cloud needs immediate processing software for fast data processing and monitoring [33].

### 2.1. Architecture of High-Speed Network

A high-speed network enables fast and effective communication between computers and the other devices possible. Applications for these networks include data centers, telecommunications, and high-performance computing.

**Figure 2:**
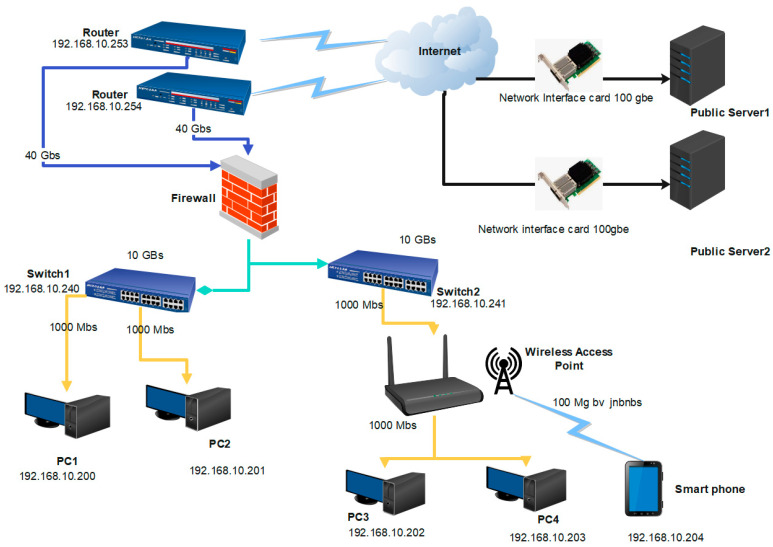
High-speed Network Architecture.

The proposed HSN architecture is presented in Figure 2. The architecture of a high-speed network typically consists of the following components: The Network Interface Card (NIC), which is a hardware component that connects the computer to the network server. The classification of its speed as bits or bytes is discussed above in Table 1. It is responsible for transmitting and receiving data between the computer and the network. The hardware that joins various computers on a network is known as a switch. It makes data transmission between the computers on the network possible. Hardware devices connect to multiple networks via a router. Fiber Optic Cable: High-speed networks typically use fiber-optic cables for transmitting data. These cables can transmit data at very high speeds over long distances. Network Operating System (NOS): The NOS is a software component that manages the operation of the network. It contains network resources, such as file sharing and printing. Another high-concern application for high-speed network is in the automotive industry. The automotive industry relies heavily on the internet, especially for procedures such as assuming two cars are going parallel. The vehicles may collide if they cannot be connected to each other. Additionally, attackers can target the vehicle’s data and hack its information. In advanced emerging auto drive, cars have several modules, such as in-vehicle networks, engine control units, body control modules, and smartphone integration modules, that provide the vehicle safety functionality [34,35]. The modules for DDoS detection must be analyzed due to the fast online data processing.

Anomaly detection is a severe issue in monitoring the services of patient due to various factors, such as patient conditions, machine error, and human ambiguity, particularly concerning disease outbreaks. For instance, many older people require routine check-ups, but cannot move in crowded places due to COVID-19 [36]. Online medical check-up applications in this era can be immensely helpful for patients with critical conditions, as they can monitor themselves from a mobile location [37]. Patient records containing essential information, such as name, age, gender, sugar level, and blood information, must be accurately monitored [38]. Real-time anomaly detection plays a vital role in tracking patients’ services and in detecting anomalies promptly. Therefore, a High-speed Network architecture can be instrumental in quickly responding to patients’ queries, as a large number of requests come in remotely [39].

### 2.2. High-Speed Network Evaluation Factors

**Figure 3:**
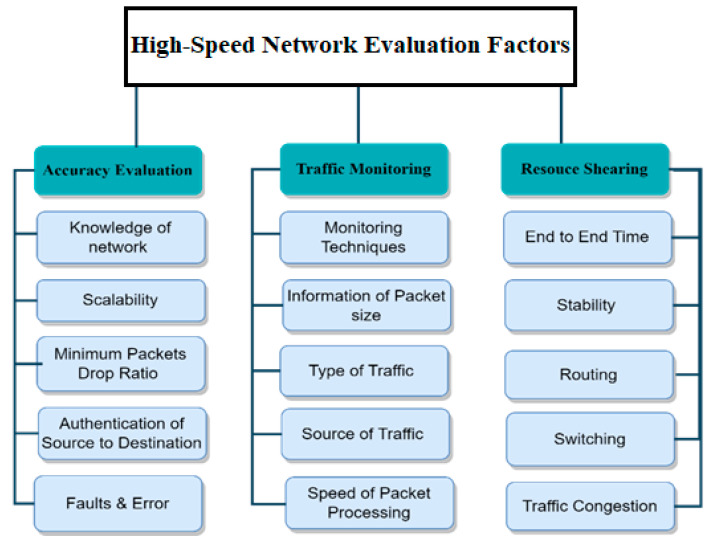
High-Speed Network Evaluation Factors.

Accuracy and speed are valuable parameters for intrusion detection and prevention systems in high-speed networks. As numerous attacks continue to emerge, accurately monitoring vulnerability using regular expression is not straightforward [40]. The taxonomy of high-speed network evaluation factors is shown in Figure 3. The proposed taxonomy is categorically divided into accuracy evaluation, traffic monitoring, and resource sharing.

An accuracy evaluation of a network depends on various factors that require different assessment options, such as knowledge of the network, scalability, minimum packet drop and receive ratio, and authentication of the source and destination, as well as fault and error analyses [41]. Mainly, these evaluations are categorized into processes, impact, outcomes, and result. Traffic monitoring, also known as network monitoring, involves the processing and analyzing of the incoming and outgoing network data using particular hardware and/or software [42]. At the basic level, different types of tools are used to monitor the network, and various parameters are checked, including information on the packet size, type of traffic (voice/I.P., control traffic, and web traffic, etc.), traffic source, and packet processing speed. During this monitoring, the data uploaded and downloaded should be displayed to the network administrator to necessitate advanced reporting. Resource sharing refers to the sharing of library resources by specific participating libraries based on cooperation. Resource sharing means making one library’s collections available to consumers. Resource sharing contains technical capabilities, end-to-end time, stability of the required resources, expertise in traffic congestion handling, and the policies of routing and switching vital to obtaining that aim.

High-speed networks are vulnerable to various security threats and attacks, compromising network resources’ confidentiality, integrity, and availability. Here are some examples of high-speed network attacks: Denial-of-Service (DoS) attacks: high-speed networks are particularly susceptible to DoS attacks, which flood the network with excessive traffic, causing it to slow down or even crash. Man-in-the-middle (MITM) attacks: in MITM attacks, an attacker intercepts the communication between two parties on the network and can eavesdrop on or modify the transmitted data. Packet sniffing: an attacker can use a packet sniffer tool to capture and analyze the network traffic, including sensitive data such as passwords, usernames, and credit card information. Malware and virus attacks: malware and viruses can infect network devices and spread quickly across high-speed networks, causing significant damage. Insider threats: high-speed networks are also vulnerable to insider threats, such as employees with malicious intent who can access confidential information and compromise network security. To protect high-speed networks from these vulnerabilities, there is a need to implement automatic security measures such as firewalls, intrusion detection and prevention systems, encryption, access controls, and regular security audits and updates [43].

## 3. Denial of Service Attacks

A denial-of-service (DoS) attack overloads a device or network, making it inaccessible. Attackers accomplish this by sending more traffic than the target can handle, causing it to fail and rendering it unable to serve its regular users. Email, online banking, websites, and any other service that relies on a targeted network or computer become vulnerable to attacks. An interconnected, distributed network of machines cause a DDoS attack, consisting of devices (e.g., IoT devices) that can be affected by malware being controlled remotely [44]. A group of bots or machines is called a botnet. A botnet can directly attack and send instructions remotely to each bot. In a botnet, affected network, or server, every bot sends request to a specified IP address, causing a DoS to regular traffic. It is challenging to separate regular traffic from attacker traffic. Common examples of DDOS attacks are UDP flooding, SYN flooding, and DNS amplification [45]. Nowadays, ultra-short DDoS attacks happen. Gcore mentioned that the average duration of a DDoS attack in 2022 is 5–10 s, and the capacity of the episode is 5 gbps in 24 h.

### 3.1. Types of DDOS Attack

**Figure 4:**
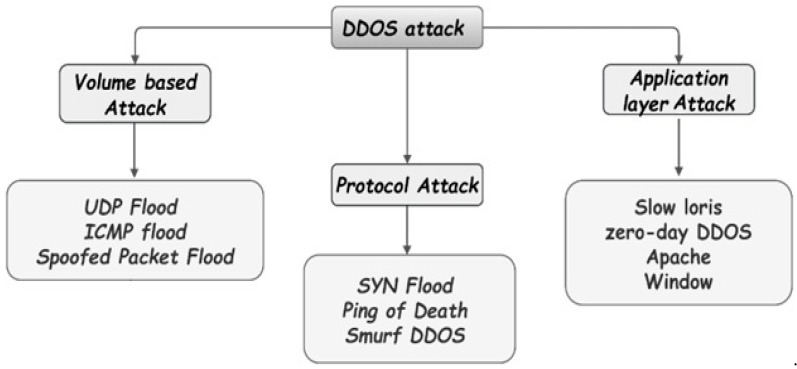
Types of DDoS attack.

There are many types of DDoS attacks, with some being grouped as a combination of multi-vector attacks, and categorizing these diverse attacks calls for other defense mechanisms. In online services, attacking the weakest link can bring down the whole network. A robust Domain Name Server will not respond when overloaded with scam requests by attackers [46]. Figure 4 shows types of DDOS attacks.

#### 3.1.1. Zero-Day Attacks

These attacks exploit previously unknown network software or hardware vulnerabilities. Since these vulnerabilities are not yet known to the vendor or the public, they can be challenging to defend against [47].

#### 3.1.2. Reflection Attacks

Similar to amplification attacks, reflection attacks use vulnerable protocols to amplify attack traffic. However, in reflection attacks, the attacker sends requests to third-party servers that then send responses back to the target network, amplifying the size of the attack traffic. It is important to note that these types of DDoS attacks can occur on both high-speed and low-speed networks, but they can be especially devastating on high-speed networks due to the large amount of traffic they can generate.

#### 3.1.3. DNS Amplification

Volumetric DDoS attacks [48], known as DNS amplification, employ an effectively enhanced reflection attack method. These attacks saturate the bandwidth by boosting the outbound data flow. The attackers issue information requests to the server that produce massive volumes of data, creating vast amounts of traffic. They then fake the reply-to address to route the information back to the server. Therefore, in a DNS amplification attack, the bad actor transmits several relatively tiny packets from numerous distinct sources in a botnet to a publicly accessible DNS server. Each of these packets makes a lengthy request, such as DNS name lookup queries. The DNS server then answers each of these dispersed queries with response packets that are several orders of magnitude larger than the initial request packet, all of which are routed back to the victim’s DNS server.

#### 3.1.4. SYN Flood

SYN flood attacks [49] bypass the three-way handshake protocol to build TCP connections between clients and servers. Typically, these connections are created by the client sending a synchronize (SYN) request to the server, and the client finishing the handshake with a final acknowledgement (ACK). SYN floods operate by rapidly delivering synchronization requests and then not replying with a final declaration from the server. The client sends a synchronize (SYN) request to the server, the server replies with an acknowledging (SYN-ACK) response, and the handshake finishes with a final acknowledgement (ACK). SYN floods operate by making these synchronization requests and leaving the server hanging by failing to react with a final declaration.

#### 3.1.5. Ping of Death

Ping-of-death attacks [49] differ from standard ICMP echo ping flood attacks. The content of the packet is maliciously engineered to induce server-side system failure. The data in a typical ping flood assault are practically irrelevant, as they are designed to overwhelm the bandwidth with sheer volume. A ping-of-death attack exploits the weaknesses in the victim’s device by sending packets that cause it to stop or break. This approach may also be applied to protocols other than ICMP, such as UDP and TCP.

#### 3.1.6. Application Layer Attack

DDoS attacks on the application layer are HTTP flood attacks [50]. Using this strategy, the offender frequently communicates with a web server or application. Web browsers generate all interactions to appear as regular user activity, but they are coordinated to consume as many server resources as feasible. The attacker’s request might range from retrieving URLs for pictures or documents via GET queries, to making server processes to a database via POST requests.

### 3.2. Identification of DDOS Attacks

**Figure 5:**
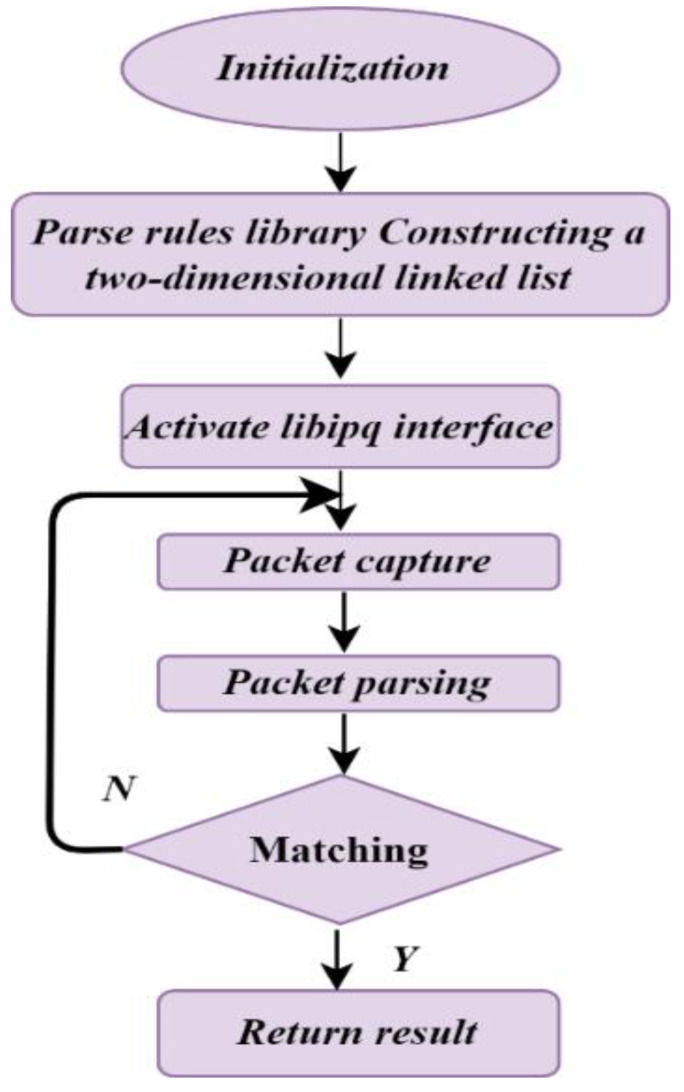
DDOS attack detection flow.

The sign of a DDoS attack is services or a site being extremely slow or unavailable. Analysis tools can point to the place where DDoS attacks occur. For instance, suspicious amounts of traffic generated from certain IP range tends to flood the network or traffic with certain behavioral profiles of device, location, and web browser information aiming at a single page or endpoint [45]. Figure 5 illustrates the basic DDoS attack detection flow as an example. There are three signs of a DDoS attack: the website seems slow to load or unavailable, the network abruptly loses internet access, and the computer becomes slow or unresponsive, indicating the presence of DDoS attacks. To detect the DDoS attack, the first step is initialization to check the system parse rules library, constructing a two-dimensional linked list. A libpq is the PostgreSQL interface for C application developers. libpq is a collection of library methods that enable apps to send requests to the PostgreSQL server and retrieve the responses. The following steps capture the packet, parse the box, and match it with the back-end server database. If the result is found, it is taken; otherwise, the package is retrieved to the libpq interface.

### 3.3. DDOS Attack Detection Techniques

In this section, the detection techniques for a DDoS attack have been elaborated. The DDoS attack attempts to load traffic on the network, application, computer, and services, forcing them to go offline. A botnet is an internet-connected device that operates more than two bots. Botnets may launch DDoS assaults, steal data, send spam, and give the attacker access to the device and its connection. The operator may manage and control a botnet using software [51]. Attackers cause botnets of the infected computer on the network to cause the service to disconnect or be unavailable. Table 3 elaborates on these DDoS attack detection techniques.

DDoS attacks require high-speed traffic analyses. The data entering various resources have a rate of approximately 28,100 Gbps, which is a massive amount of data when it comes to a NIC 100 Gbps of command line. A Socketbase packet analysis is not suitable for fast data processing. Instead, the express data path is used, which is a sub-program of the Barkley packet filter that utilizes different aspects and an extended Barkley packet filter [70,71]. Currently, various tools are used to detect and prevent DDoS attacks on networks [72]. These tools monitor the event logs from various sources to detect and prevent DDoS activities. Table 4 describes the DDoS attack prevention tools.

## 4. DDOS Attack Detection in High-Speed Network

An open-source Intrusion Detection System, Snort and Suricata [78], explains how to evaluate the drop rates and accuracy rates in a 100 Gbps network using their comparison and benchmarks [79]. This evaluation includes the usage of system resources, packet processing speed, packet drop ratio, and detection accuracy. However, a shortcoming of this study is that it does not consider the extensive data on the network. Another model proposed by [80], the Very Long Short-Term Memory (VLSTM) learning model, deals with the challenges of high dimensionality and unfairness. Its performance in experiments has resulted in using the UNSW-NB15 open dataset. A study presented reconstruction loss, classification loss, and divergence loss. However, anomaly detection tasks are still challenging for imbalanced data.

An Extended Barkley packet filtering (eBPF) and express data path are presented by M. A. Vieira [81] to introduce new technology for packet filtering and provide an example of a standard procedure of these technologies. The XDP program is written in the C or P4 languages, and the instructions are processed through the kernel and other programmable devices, such as a smart network interface card. This work mainly focuses on network monitoring, traffic analysis, load balancing, and system profiling. Moreover, the authors dealt with the high speed of network data but did not address the packet drop ratio. In given Table 5, the studies of the recent five years are categorized based on different parameters such as year, article reference, main features, advantages, and weaknesses.

The researchers in [84] processed a massive amount of network traffic with a verification technique that checked the reliability based on the classifier’s outcomes. The Big-Flow classification model is adjusted once suspect packets are found. The focus of this study is to deal with the network traffic dataset, but it does not consider the packet drop ratio. According to [59], the DDoS detection schema has numerous traffic functions. This scheme generates precise per-subnet alarms implemented in the data plane without external controllers, allowing for tight control loops. The findings include accurate detection relying on a realistic attack using accessible traces. It deals with incoming flows and the packet symmetry ratio observed per secured sub-network. The express data path is a suitable framework for DDoS protection and creating a novel scheme to prevent cyber threats. Nevertheless, it features packet rates of 1–2 Mpps for 10 Gigabit links not more than 10 Giga bit. Table 6 shows the comparison of an irregular traffic pattern.

A Linux subsystem is capable of tracking containerized user-space programs for Inter ledger connectors, with the ability to control the software stack in development [43]. The tests investigated and evaluated the tool landscape developed to assist eBPF in this project. This project does not show the end-to-end view of a distributed system. In addition, HTTPS encrypted traffic is analyzed to determine the user’s operating system and track the user’s local explorer and other methods, resulting in a 20,000 dataset example with a 96.06% classification accuracy [96]. The traffic analysis technique, which employs SSL/TLS, is a powerful method. The attacker can use statistics to identify the user’s operating system.

A data distributed control system (DDCS) can be used for data-driven cyber security, social, and internet traffic analyses, cyber security data collection, cyber security feature engineering, and simulation [85]. The DDCS shows a strong link among data, models, and methodology while reviewing the key recent works in Twitter spam detection and I.P. traffic classification. However, this work does not mention high-speed data.

The research in [80] suggested a new malicious classification scheme based on the Long Short-Term Memory (LSTM) model. Data annotation for effective traffic classification can result in network loops and bandwidth issues. The selection of LSTM makes it accurate. In a DDoS, the detection schema has numerous traffic functions [59]. These features are known as formal DoS parameters, such as the arriving flow pattern and packet symmetry levels observed per secured sub-network. In [97], a full-packet capture in (FPC-NM) systems in 20 Gb/s was developed and deployed. A nanosecond timestamp was used in the FPC-NM system, significantly boosting the accuracy of a security incident retrospective analysis.

Implementing the FPC-NM system achieves a 17 Gb/s throughput with a connection of 160,000, experiencing zero packet loss. These parameters encompass packet reception, nanosecond timestamping, load balancing, preprocessing packets, application layer protocol analysis, data packet storage, and log management. By utilizing LZ4 compression, the system achieves real-time compression and storage efficiency at 10 Gb/s, but up to 40 Gb/s. However, it does not support 70 Gbps and 100 Gbps. As industry and research institutions are installing 100 Gbps networks to meet data transfer demands, high-speed networks are becoming more common, leading to significant technical challenges. An Intrusion Detection System cannot efficiently handle network activities with high rates of traffic monitoring and packet drop ratios, which directly affects the detection accuracy. This paper [87] provides a detailed explanation of the open-source IDS, namely, Snort and Suricata, with comparative parameters in a 100 Gb/s network.

**Figure 6:**
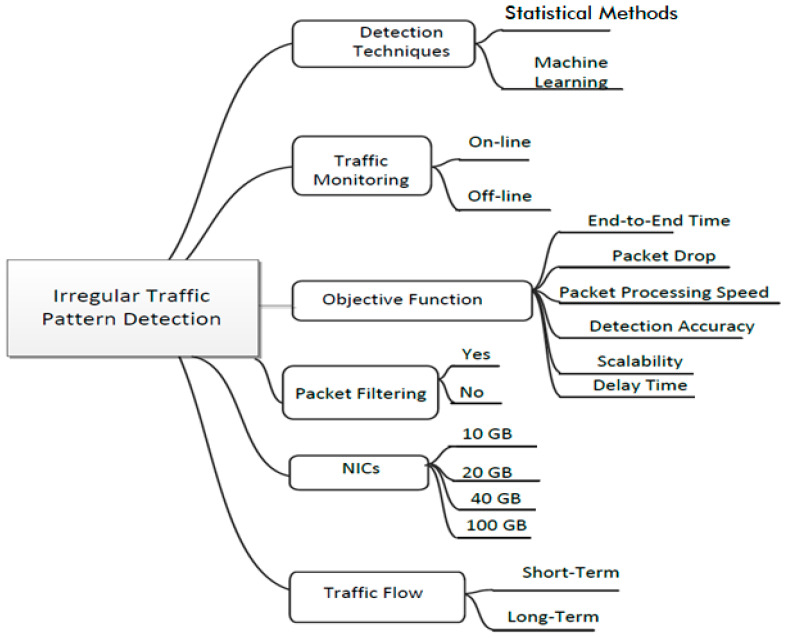
Irregular traffic pattern detection.

A low-rate DDoS attack detection method (LDDM) using a multidimensional sketch structure and network flow measuring allows for a reduction in the data storage cast and improves the detection accuracy [65]. The measurements depend on the daubechies four wavelets transform to calculate each sketch’s energy percentage. This approach differentiates between the regular and attack traffic. The LDDM is used to evaluate low-rate DDoS attack datasets, but a high-rate DDoS attack is not considered. Figure 6 shows different irregular traffic pattern detection.

The architecture in [98] allows for network operators to estimate the flow size of encrypted data at multi-Gbps line rates using samples and sketching mechanisms. It also helps in understanding the behavior of VPN-buffered traffic. The implementation shows a 99% accuracy of the service provider on 6000 tracks for three key factors. Evaluation studies depend on the track time and starting point, achieving more than a 90% precision for the content classification of a given service provider in the best case. The examiner presents the time path’s performance (XDP). eBPF is used for XDP to process incoming traffic before allocating kernel data structures, which improves the performance. The second case study uses eBPF to set up socket-level application-specific packet-filtering options. To eliminate errors and produce a custom binary for a specific network function, Packet-Mill boosts the throughput (up to 36.4 Gb/s—70%) and reduces the latency (up to 101 Gb/s—28%) without continuing unnecessary packet processing at 100 Gb/s. However, new packets arrive 10 times faster than main memory access times while utilizing only one processor core [91]. Apache storm used the Netty communication component [98], a TCP/IP protocol stack applied for an asynchronous server, and a client framework that decreased efficiency due to context switching and memory copying. It increased the IP over the InfiniBand communication mode on the CPU load. With the aid of remote direct memory access (RDMA) technology, the scheme implementation can reach up to five times faster than IPoIB and ten times faster than Gigabit Ethernet when tested on Mellanox QDR Cards (40 Gb/s). Additionally, this approach considerably reduces the CPU burden and boosts the system throughput.

Comma-separated values (CSV) [97] are a frequently used data interchange format. Concerning format, all industries’ potent databases and stream processing of frameworks have utilized CSV as an input. The speed of input or output hardware poses significant challenges due to advanced input or output gadgets such as InfiniBand NICs and NVMe SSDs, with transfer rates of 100 Gb/s and higher. This article aims to increase the input speed of CSV with the help of graphics processing unit GPUs. A new parsing strategy is created that simplifies the control flow, while correctly handling context-sensitive CSV features such as quotes. In Table 7, the articles have been studied and categorized based on their main features, advantages, and drawbacks. This section defines the thematic taxonomy of the characterization and classification of the irregular traffic pattern schemes on high-speed data networks, in order to achieve the following objectives: end-to-end time, packet drop, packet delay time, scalability, packet processing speed, and detection accuracy. The stated studies are categorized based on six characteristics: (i) detection techniques, (ii) traffic monitoring, (iii) NICs, (iv) traffic flow, (v) traffic filtering, and (vi) objective function.

Network traffic monitoring is a task to ensure that the operation of a network performs smoothly. When any unusual packet comes on the network, the Network Traffic Monitoring Tool (NTMT) [105] captures that packet. Generally, NTMT observes all incoming and outgoing packets on the network. Detection accuracy implies the agreement between the actual and detection values. The exact value is unknown in several cases, but is compared with the standard. Accuracy is a ratio of the nearest value to the real value, which is the result. Scalability is a characteristic of a system, model, or function that elaborates on the ability to manage the workload. In the scalability test, many parameters are included, such as throughput, memory usage, CPU usage, network usage, and response time. Delay time is the time between the source signal and its echo. The most uncomplicated delay effect is a single repeat. The minimum delay is counted as 30 and 100 ms to create a slap-back echo, while longer delay times produce a more distant echo.

## 5. Issues and Challenges

**Figure 7:**
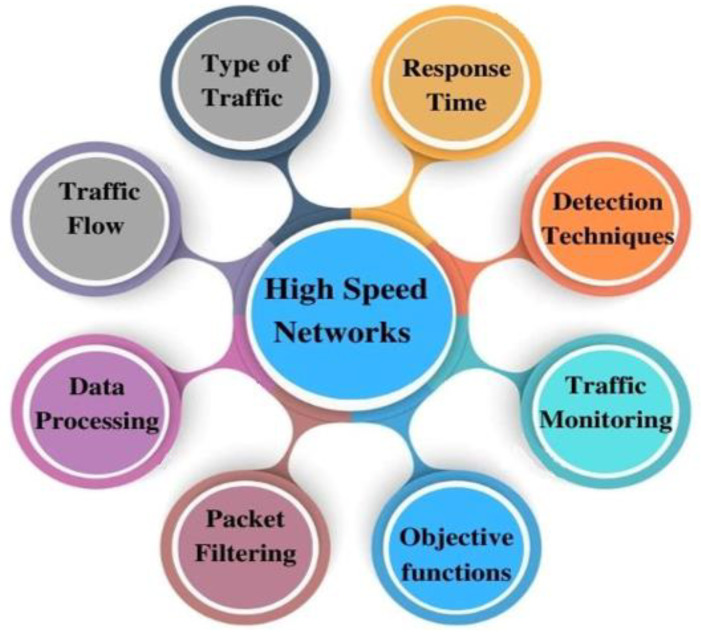
High-speed network issues.

This section explains the open research issues and challenges concerning HSNs. HSN issues are characterized based on factors such as data processing, traffic monitoring, packet filtering, traffic filtering, packet drop, response time, and packet size, as presented in Figure 7. Moreover, Table 8 shows the types of DDoS attacks with respect to the different parameters in high-speed networks, as highlighted in Figure 7. Table 8 comprises four horizontal sections (year, reference, DDoS attack, and network issues), and the existing works are stated vertically. The existing literature is presented based on publication year in the first two columns. DDoS attacks are generally categorized into three types: application-layer attacks, volume-based attacks, and protocol attacks. In this section, a detailed explanation is provided by dividing the existing literature with respect to the DDoS attack categorization [106].

Application-layer attacks occur at the seventh layer of the Open Systems Interconnection (OSI) model. First, an attacker establishes a connection with the target. After establishing a link, the attacker exploits the resources to jam the traffic by inundating the system with excessive requests. This scenario exemplifies HTTP floods and Domain Name System floods [107]. Volume-based attacks are launched against individual targets, and commonly target service providers (SPs). The attacker monopolizes the network’s bandwidth and inundates the server with a barrage of packets to overwhelm it. Examples include TCP floods and UDP flood attacks [56]. Protocol attacks involve sending a flood of traffic with false data to the server, causing data overflow, server crashes, and rendering the resources of the server unavailable. For instance, Border Gateway Protocol (BGP) and ping of death [108].

Detecting attacks, such as DoS and DDoS, is very complex to handle in a high-speed network due to the fast packet processing required. The flow of data from numerous IoT devices occurs at a high speed, making it challenging to monitor and classify traffic accurately. DDoS attacks, in particular, generate vast amounts of traffic from multiple devices to overwhelm the server resources, and the data processing must be extremely fast. Consequently, network monitoring struggles to keep up with the high-rate traffic and may result in packet drop and a reduced scalability [109]. Various software-defined network techniques have been developed to mitigate DDoS attacks [12,52], including content delivery networks that improve performance while countering DDoS attacks [60]. In recent studies [110,111], XDP (express data path) and eBPF (extended Berkeley packet filter) have been utilized for high-performance data analysis paths on networks, such as in Netmap [109] and Data Plane Development Kit (DPDK) [71,112]. These techniques have proved valuable in processing data at the application layer. Despite the numerous frameworks proposed for packet classification and network traffic analysis, the speed of packet processing speed still requires improvement, especially when dealing with specialized hardware [97]. Figure 7 highlights the discussion on the challenges of high-speed networks.

The term objective function is explained as end-to-end delay (EED) or one-way delay (OWD), both of which refer to calculating the time duration of a packet’s transfer from the source to destination. EED is commonly used for IP network monitoring, but it contrasts with round-trip time (RTT), which calculates the time for a packet to travel from the source to destination and back. Packet drops or losses can occur due to network traffic congestion, hardware issues, or software viruses. In computer networks, a DoS attack can manifest in different forms, such as packet drop attacks or black hole attacks. Detecting and preventing packet drop attacks is much harder, especially in high-speed data transfers on a network. The speed of the packet processing is directly related to the rate at which the data or information are processed across the network, and a higher performance of the network interfaces requires faster packet-processing speeds. High-speed routers act as forwarding engines, allowing them to handle the growing internet traffic demand without slowing down. To accommodate multi-terabit IP routers, data centers also require switches that can sustain a throughput of a hundred Gbps and multiple Tbps.

When a large packet is transmitted on a 100 gigabit lane network, the number of packets per second should decrease. The packet drop ratio is high when small packages are sent over this network interface card. The snort performs tests accurately on the data processing, detecting malicious activity on the network at an average speed. It focuses on high-speed traffic, inspecting incoming packets for potential threats. The ratio of dropped packets is higher for malicious traffic. Custom snort rules have been tested on 1 Gbps NICs by using different attack generators [111]. Despite various security technologies, compressive techniques to solve DDoS issues remain challenging. The researcher in [113] faced some challenges while trying to detecting DDoS attack solutions, as described below. High-rate flooding attacks are achieved by deploying open-source DDoS tools on the attacker’s machine during a DDoS attack, maintaining the server resources until the session expires [114]. In a high-speed network, fast monitoring tools are utilized when a DDoS attack occurs, considering factors such as the number of connections, attack force, and impulsive protocols. The DDoS prevention system works keenly to stop malicious data from distributed nodes, grouping the malicious in the data-processing system. A bottleneck at a buffer queue creates a link with the attacking packets when the attack rate is high. It is impossible to decide which resources to allocate for the server without employing a classification approach [115]. Datasets for DDoS prevention systems analyze the standard of testing for real-time implementation. However, the authors of [116] failed identify the actual standard dataset for the testbed. The DDoS signature base attacks enlist all the feasible features of signatures in real-time [117].

Application-layer DDoS attacks prevent applications from providing specific content to users. Commonly targeted web servers and applications include Session Initiation Protocol (SIP) voice servers and BGP [65]. Hence, DDoS attacks are often initiated by intelligent clients, and they typically do not spoof IoT devices. There are seven types of application-layer attacks: slow loris, slow post, slow read, HTTP flooding, low and slow attack, large payload post, and Mimicked User browsing. Conversely, HTTP flood attacks involve a botnet, where the attacker sends numerous requests to overwhelm the target [118,119]. Volume-based DDoS assaults concentrate on the server’s bandwidth. Attackers frequently craft large requests with small sizes to overload the server, leading to crashes or instability. Some examples of Volumetric DDoS attacks at layer 3 are: a DNS NX domain flood, DNS Query flood, GRE attack, Internet Control Message Protocol type (ICMP) flood, and IP/ICMP fragmentation garbage flood [119]. A sign of a volumetric DDoS attack is a higher bandwidth with ongoing data, reaching up to 100 Gb or terabits/second. A volumetric attack is easy to generate by applying easy amplification techniques. The sheer quantity of the traffic generated by the attack can entirely block access to end resources (a website or a service). The magnitude of the attack is typically measured in bits or packets per second [120]. These attacks expose weaknesses in layer 3 and the four protocol stack. Protocol attacks consume the processing capacity of the attack target or intermediate critical resources, causing service disruptions. For instance, SYN floods and ping of death are examples of protocol-based attacks. A TCP SYN flood is a protocol-based attack where a sequence of TCP SYN requests is sent directly to the target server, making it unresponsive. In a recent DDoS outage, an application-layer attack involved TCP SYN floods targeting port 53 of Dyn’s DNS servers [78].

## 6. Conclusions and Future Directions

This paper primarily classified DDoS attacks and the types that can occur in a high-speed network. The DDoS issue is a rapidly growing problem. This research also examined the various existing solutions for detecting DDoS attacks, including traceback mechanisms, which are classified into proactive and reactive approaches, packet marking such as PPM and DPM, and application layer protocol analyses to improve the detection accuracy in terms of the monitoring and filtering of affected data packets using an express data path. This article detailed the growing differences between regular and irregular traffic in terms of DDoS attacks. Additionally, the high-speed vulnerabilities, problems, and challenges of the network layer for maximum packet processing were also explored. High-speed packet processing, detecting, and preventing a DDoS is difficult, and the packet drop ratio is high. DDoS mitigation in high-speed networks is progressing quickly, and researchers are developing efficient and innovative solutions. The open issues and challenges discussed above provide an ideal picture for future directions regarding DDoS detection in high-speed networks. Different studies have been proposed to process data quickly based on 100 Gbe Network interface cards. However, these studies often overlook the packet drop ratio and the data management at the Kernal level using 100 Gbe.

## Figures and Tables

**Table 1:** Packet speed category.

Speed	Bits/s	Bytes/s	Maximum Packet/s	Type of Traffic	
				Low Speed	High Speed
10 Mbps	10 × 10^5^	125 × 10^4^	14,881	✔	✗
100 Mbps	10 × 10^6^	125 × 10^5^	148,810	✔	✗
1 Gbps	10 × 10^7^	125 × 10^6^	1,488,095	✔	✗
10 Gbps	10 × 10^8^	125 × 10^7^	14,880,952	✔	✗
100 Gbps	10 × 10^9^	125 × 10^8^	148,809,524	✗	✔

**Table 2:** Network traffic type and characteristics.

Characteristic	Traffic Type		
	Voice	Video	Data
Real-time	Yes	Yes	No
TCP/UDP	UDP	UDP	TCP
Packet Delay	Sensitive	Sensitive	Insensitive
Packet Drop	Sensitive	Sensitive	Insensitive
Benign/Greedy	Benign	Greedy	Both
Smooth/Busty	Smooth	Busty	Both
Mobility	Yes	Yes	Yes

**Table 3:** Categorize the studies based on the DDoS detection techniques.

Year	DDOS Attacks	DDOS Detection Techniques	Articles
2018	Application, volume base	Support vector machine (SVM), PCA-KNN, fuzzy logic G.A.	[4,7,52,53,54]
2019	Application layer	Entropy, signature base, support vector machine (SVM), bat algorithm.	[50,55,56]
2020	Application layer	Naïve Bayesian system, support vector machine, decision tree, genetic algorithm and fuzzy logic, a spatial and temporal neighbor.	[45,57,58,59,60,61]
2021	Application layer, transport layer	Long Short-Term Memory (LSTM), low rate, allow listing and block-listing, rate limiting, Random Forest, multi-layer Perceptron, fast all-packets-based, Divide and Conquer, token-bucket mechanism.	[51,62,63,64,65,66,67]
2022	Application layer	Information Gain, Random Forest, LTSM, low rate, SVM, R.F., L.R., KNN, D.T., NB, DPS, CPU time, PGA.	[12,16,17,18,68,69]

**Table 4:** Comparison of DDoS attack Prevention Tools.

Ref#	Tools	Attacks	Outcome
[73]	SolarWinds SEM Tool	It is a software to detect and prevent the DDOS attack	SEM’s mechanism for maintaining logs and events that helpful for post-breach investigations and DDoS mitigation.
[74]	HULK	It produces single and unclear traffic	It fails to hide identity. It can block traffic via HULK.
[75]	Tor’s Hammer	Apache and IIS server	Tors hammer implements a DoS attack using a slow POST attack and HTML posts at a slow rate within the same session (actual rates of random selection is 0.5–3 s).
[76]	Slow loris	DDOS attacks on HTTP traffic	To prevention of DDOS attacks, HTTP traffic data sent to the target server.
[77]	Low Orbit Ion Cannon (LOIC)	DOS attacks on UDP, TCP, and HTTP traffic	LOIC checks the network stress and malware authors create the virus.
[78]	XOIC	DoS attack on Internet Control Message Protocol	In XOIC, this is a tool to block the attack.

**Table 5:** The studies of DDoS attacks in a high-speed network.

Year	Article	Main Features	Advantages	Weakness
**2018**	[52]	The author proposed a three-layer module DDOS attack identification, delivery module flow table, and traffic identification	The applied SVM to DDoS traffic identification.	The flow table delivery module is needed to improve.
**2018**	[53]	For DDoS mitigation, traffic MoonPol	High-performance packet processors used by policers like DPDK.	The small number of packets that randomly falls into subnets of limited ranges.
**2018**	[82]	A non-parametric methodology in the data stream	Statistical based, distance-based detection.	Not optimized to find anomalies.
**2018**	[83]	The present Time Path’s performance (XDP)	Just-in-time (JIT), kernel hook.	It is needed to capture the packets at a high data rate.
**2019**	[55]	Detection of DDoS attacks at the application layer	Analysis about HTTP DDOS monitoring, detection, mitigation, and prevention.	This study does not consider high-speed networks.
**2019**	[84]	The Big-Flow classification model	Network traffic dataset, scalable.	Does not consider the packet drop ratio.
**2019**	[85]	Data-driven cyber-security is used for internet traffic analysis	Cybersecurity, network traffic analysis, machine learning (ML), and social scam detection.	Research is required for extensive data networks, domain knowledge of traffic monitoring.
**2019**	[86]	To build the rule of DDOS mitigation in smart NICs on offloading the edge server	Smart NICs can help mitigate the network load on congested servers	Smart NICs reduce the effectiveness of server resources.
**2020**	[58]	Extended Berkeley packet filter and express data path	Packet filtering	Does not consider the packet drop ratio.
**2020**	[59]	DDoS detection schema	Incoming flows, packet symmetry ratio.	Does not consider delay time
**2020**	[80]	A VLSTM learning model	Reconstruction loss, classification loss, and divergence loss.	Anomaly detection tasks are still challenging for imbalanced data.
**2020**	[81]	Extended Barkley packet filtering (eBPF) and express data path	Network monitoring, network traffic analysis.	Does not deal with big data.
**2020**	[87]	Open-source Intrusion Detection System: Snort and Suricata	Speed of packet processing, packet drop ratio, the accurateness of detection.	Does not consider the extensive data on the network.
**2020**	[88]	Experiment with a Linux subsystem to track containerized user-space programs	Interpledge, eBPF, Profiling, Tracing.	It is not created for an end-to-end view of a distributed system.
**2021**	[64]	To suggest a new malicious classification scheme based on the Long Short-Term Memory (LSTM) model	LSTM, accuracy, throughput. Traffic classification, artificial intelligence, malicious traffic.	Using upcoming learning strategies, the metric selection for LSTM can be made accurately.
**2021**	[65]	This article proposed a new Learning Design Discussion Model (LDDM)	Lower false positive and false negative rates. DDoS attacks.	Still improve the detection accuracy on high-speed data 100 Gbps network.
**2021**	[79]	To estimate the flow size of encrypted data at multi-Gbps line rates	Deep Packet Inspection, multi-Gbps line, VPN-buffered traffic.	Still improve the detection accuracy on high-speed data 100 Gbps network.
**2021**	[89]	Estimate the overall number of unique components or different k-constant items in a flow across various traffic measurement	Filter out duplicates, sample the elements, and store the sampled traffic data in off-chip memory using it on memory.	Cannot detect distributed denial of service attacks and scanners.
**2021**	[90]	In this paper, we develop and deploy a full-packet capture in (FPC-NM) systems	Packet reception, data packet storage, and log management.	Up to 40 Gbs, 70 Gbs, and 100 Gbs are not included.
**2021**	[91]	To eliminate errors and produce a custom binary for specific network	Code-optimization approaches.	Does not continue the packet processing at 100 Gbps.
**2022**	[68]	The algorithm monitors the CPU time used by every connection and the statistical method used for attack detection	System Calls information is container-based on Linux eBPF at the host level.	This algorithm considers only Dos attacks, not DDoS attacks.
**2022**	[69]	Signature-based techniques for DDoS mitigation and utilization of Packet generation algorithms (PGA) for attack execution	Full-fledged IDS/IPS solutions like Snort Suricata.	To unlock the full potential of eBPF and XDP (cross-compiling, modularity).
**2022**	[92]	NetFPGA SUME approach used for packet filtering and mitigation of volumetric DDOS attack	Packet filtering has been performed in HSN using a single core of CPU.	A 100 Gbit/s data path provides an excellent testing environment.
**2022**	[93]	HARNESS schedule and serve as control plane USRs in terms of delay tolerant and delay-sensitive to authenticate H.A. services.	XDP and eBPF use for coherent and optimized end-to-end working.	Does not consider the packet drop ratio.

**Table 6:** The comparison of irregular traffic pattern detection.

Article	Detection Technique	Traffic Monitoring	Packet Filtering	NICs	Traffic Flow
[58]	Kubernetes	Offline	Yes, eBPF/XDP	NA	NA
[62]	Kernel JIT/translator	Offline	Yes (eBPF/XDP)	Smart NICs	NA
[80]	Vibrational LSTM	Online	No	NA	Long/Short-Term
[81]	P4 language	Online	Yes, eBPF/XDP	Smart NICs	NA
[84]	Big Flow	Online	No	10 GB/s	Big flow
[87]	Snort, Suricata	Offline	Yes	100	Long/Short-Term
[88]	PMDA modules	Offline	Yes (BPF)	-	-
[94]	hXDP	Online	Yes (eBPF)	100 Gbps	Big flow
[95]	XDP	-	Bpf, eBPF, XDP	-	Long/Short Term

**Table 7:** Objective function of irregular traffic pattern.

Objective Functions	Description	References
End to End time	This one path direction calculated as time from source to destination.	[99]
Packet drop	Packet drops or packet loss can occur during network traffic congestion, hardware problems, and software viruses.	[100]
Packet processing speed	The rate of data flow is across the network.	[101]
Detection accuracy	Detection accuracy implies the agreement between the actual and detection values.	[102]
Scalability	Scalability is a system, model, or function characteristic to elaborate the ability to manage the work.	[103]
Delay time	Calculating the time duration of a packet transferring from source to destination.	[104]

**Table 8:** High-speed network issues.

Year	Reference	DDOS Attack	Network Issues						
			Data Processing	Traffic Monitoring	Packet Filtering	Traffic Flow	Packet Drop	Response Time	Packet Size
2018	[4]	Application, volume base	✔	✔	✗	✔	✗	✔	✗
	[7]		✔	✔	✔	✔	✗	✔	✔
	[52]		✔	✔	✔	✔	✗	✗	✗
	[53]		✔	✗	✔	✔	✔	✗	✔
	[54]		✗	✔	✗	✔	✗	✗	✗
2019	[55]	Application layer	✔	✔	✗	✔	✗	✔	✔
	[56]		✗	✔	✔	✔	✗	✔	✔
	[50]		✔	✔	✗	✔	✗	✗	✔
2020	[57]	Application layer	✗	✔	✔	✔	✗	✔	✗
	[58]		✔	✔	✔	✔	✗	✗	✗
	[45]		✗	✔	✗	✔	✗	✗	✗
	[59]		✔	✔	✔	✔	✗	✔	✗
	[60]		✔	✔	✔	✔	✔	✔	✔
	[61]		✔	✔	✗	✗	✗	✔	✗
2021	[51]	Application, transport layer	✔	✔	✗	✔	✗	✔	✔
	[62]		✔	✔	✗	✗	✗	✔	✗
	[63]		✔	✔	✔	✗	✗	✔	✗
	[64]		✔	✔	✔	✔	✗	✔	✗
	[65]		✔	✔	✔	✔	✗	✔	✔
	[66]		✗	✗	✗	✔	✗	✔	✔
	[67]		✔	✗	✔	✔	✔	✔	✔
2022	[12]	Application layer	✔	✗	✗	✔	✔	✔	✔
	[16]		✔	✔	✔	✔	✔	✔	✔
	[17]		✔	✔	✔	✔	✔	✔	✔
	[18]		✗	✔	✔	✔	✔	✔	✗
	[69]		✔	✔	✔	✗	✔	✔	✗

## Data Availability

Not applicable.

## Abbreviations

BPF	Berkely Packet Filter
CPS	Cyber–Physical System
DDCS	Data Distributed Control System
DDOS	Distributed Denial of service attack
DNS	Domain Name System
DOS	Denial of service attack
DPDK	Data Plane Development Kit
eBPF	Extended Berkely Packet Filter
EED	End-to-end delay
GBS	Giga bit per second
GPS	Global positioning system
HSN	High-speed Network
HTTP	Hypertext Transfer Protocol
QoS	Quality of Service
ICMP	Internet Control Message Protocol
IDS	Intrusion detection system
IOT	Internet of Things
IP	Internet Protocol
IPoIB	Internet Protocol over InfiniBand
LDDM	Learning Design Discussion Model
MCC	Mobile Cloud Computing
KNN	K-Nearest Neighbor
NB	Naive Bayes
ML	Machine learning
NIC	Network interface card
NTMT	Network Traffic Monitoring Tool
OWD	One-Way delay
IDS	Intrusion Detection System
RTT	Round-Trip Time
SQL	Structured Query Language
SSL	Secure Sockets Layer
TCP	Transmission Control Protocol
TLS	Transport Layer Security
MC	Mobile Computing
UDP	User Datagram Protocol
URLs	Uniform Resource Locator
VLSTM	Very Long Short-Term Memory
XDP	Express data path

## References

[1] Haseeb-Ur-Rehman R.M.A., Liaqat M., Aman A.H.M., Ab Hamid S.H., Ali R.L., Shuja J., Khan M.K. (2021). Sensor cloud frameworks: State-of-the-art, taxonomy, and research issues. IEEE Sens. J..

[2] Chaâri R., Ellouze F., Koubâa A., Qureshi B., Pereira N., Youssef H., Tovar E. (2016). Cyber-physical systems clouds: A survey. Comput. Netw..

[3] Cisco U. (2021). Cisco annual internet report (2018–2023) white paper. Acessado Em..

[4] Li Q., Meng L., Zhang Y., Yan J. (2018). DDoS attacks detection using machine learning algorithms. International Forum on Digital TV and Wireless Multimedia Communications.

[5] Yusof A.R.a., Udzir N.I., Selamat A. (2019). Systematic literature review and taxonomy for DDoS attack detection and prediction. Int. J. Digit. Enterp. Technol..

[6] Cheng J., Xu R., Tang X., Sheng V.S., Cai C. (2018). An abnormal network flow feature sequence prediction approach for DDoS attacks detection in big data environment. Comput. Mater. Contin..

[7] Singh K.J., Thongam K., De T. (2018). Detection and differentiation of application layer DDoS attack from flash events using fuzzy-GA computation. IET Inf. Secur..

[8] Akbari E., Tabatabaei S.M., Yazdi M.B., Arefi M.M., Cao J. (2023). Resilient backstepping control for a class of switched nonlinear time-delay systems under hybrid cyber-attacks. Eng. Appl. Artif. Intell..

[9] Zheng A., Huang Q., Cai D., Li J., Jing S., Hu W., Wu J. (2019). Quantitative assessment of stochastic property of network-induced time delay in smart substation cyber communications. IEEE Trans. Smart Grid.

[10] Ganesh P., Lou X., Chen Y., Tan R., Yau D.K., Chen D., Winslett M. (2021). Learning-based simultaneous detection and characterization of time delay attack in cyber-physical systems. IEEE Trans. Smart Grid.

[11] Ullah S., Choi J., Oh H. (2020). IPsec for high speed network links: Performance analysis and enhancements. Future Gener. Comput. Syst..

[12] El Sayed M.S., Le-Khac N.-A., Azer M.A., Jurcut A.D. (2022). A Flow Based Anomaly Detection Approach with Feature Selection Method Against DDoS Attacks in SDNs. IEEE Trans. Cogn. Commun. Netw..

[13] Papalkar R.R., Alvi A.S. (2022). Analysis of Defense Techniques for DDOS Attacks in IoT—A Review. ECS Trans..

[14] Naqvi I., Chaudhary A., Kumar A. (2022). A Systematic Review of the Intrusion Detection Techniques in VANETS. TEM J..

[15] Almansor M., Gan K. (2018). Intrusion detection systems: Principles and perspectives. J. Multidiscip. Eng. Sci. Stud..

[16] Rios V.D.M., Inacio P.R., Magoni D., Freire M.M. (2022). Detection and Mitigation of Low-Rate Denial-of-Service Attacks: A Survey. IEEE Access.

[17] Gupta B., Chaudhary P., Chang X., Nedjah N. (2022). Smart defense against distributed Denial of service attack in IoT networks using supervised learning classifiers. Comput. Electr. Eng..

[18] Ennemoser F.J., Sattler P., Zirngibl J. State of the Art of DDoS Mitigation Techniques. Proceedings of the Seminar IITM WS 21/22.

[19] Falk H. (1997). Building local networks with hubs. Electron. Libr..

[20] Davis E.L. (1995). Fast ethernet: 100BaseTX and 100BaseT4 network interface adaptor architectures. Emerging High-Speed Local-Area Networks and Wide-Area Networks.

[21] Adrian D., Durumeric Z., Singh G., Halderman J.A. Zippier ZMap: Internet-Wide Scanning at 10 Gbps. Proceedings of the WOOT 8th USENIX Workshop on Offensive Technologies.

[22] Arashloo M.T., Lavrov A., Ghobadi M., Rexford J., Walker D., Wentzlaff D. Enabling Programmable Transport Protocols in High-Speed NICs. Proceedings of the NSDI, 17th USENIX Symposium on Networked Systems Design and Implementation.

[23] Naeem M., Jamal T., Diaz-Martinez J., Butt S.A., Montesano N., Tariq M.I., De-la-Hoz-Franco E., De-La-Hoz-Valdiris E. (2022). Trends and future perspective challenges in big data. Advances in Intelligent Data Analysis and Applications.

[24] Zubaroğlu A., Atalay V. (2021). Data stream clustering: A review. Artif. Intell. Rev..

[25] Linguaglossa L., Rossi D., Pontarelli S., Barach D., Marjon D., Pfister P. (2019). High-speed data plane and network functions virtualization by vectorizing packet processing. Comput. Netw..

[26] Alghawli A.S. (2022). Complex methods detect anomalies in real time based on time series analysis. Alex. Eng. J..

[27] Srikanth G.U., Jaffrin L.C. (2022). Security Issues in Cloud and Mobile cloud: A Comprehensive Survey. Inf. Secur. J. A Glob. Perspect..

[28] Shamshirband S., Fathi M., Chronopoulos A.T., Montieri A., Palumbo F., Pescapè A. (2020). Computational intelligence intrusion detection techniques in mobile cloud computing environments: Review, taxonomy, and open research issues. J. Inf. Secur. Appl..

[29] Katal A. (2022). Security and Privacy in Mobile Cloud Computing. Mathematical Modeling for Intelligent Systems.

[30] Kalra V., Rahi S., Tanwar P., Sharma M.S. (2022). A Tour Towards the Security Issues of Mobile Cloud Computing: A Survey. Emerging Technologies for Computing, Communication and Smart Cities.

[31] Motwani A., Shukla P.K., Pawar M. (2022). Ubiquitous and smart healthcare monitoring frameworks based on machine learning: A comprehensive review. Artif. Intell. Med..

[32] Desai F., Chowdhury D., Kaur R., Peeters M., Arya R.C., Wander G.S., Gill S.S., Buyya R. (2022). HealthCloud: A system for monitoring health status of heart patients using machine learning and cloud computing. Internet Things.

[33] Dahunsi F.M., Idogun J., Olawumi A. (2021). Commercial cloud services for a robust mobile application backend data storage. Indones. J. Comput. Eng. Des. (IJoCED).

[34] Lin X., Ma B., Wang X., He Y., Liu R.P., Ni W. Multi-layer Reverse Engineering System for Vehicular Controller Area Network Messages. Proceedings of the 2022 IEEE 25th International Conference on Computer Supported Cooperative Work in Design (CSCWD).

[35] Jan S.A., Amin N.U., Shuja J., Abbas A., Maray M., Ali M. (2022). SELWAK: A secure and efficient lightweight and anonymous authentication and key establishment scheme for IoT based vehicular ad hoc networks. Sensors.

[36] Pranggono B., Arabo A. (2021). COVID-19 pandemic cybersecurity issues. Internet Technol. Lett..

[37] García L., Tomás J., Parra L., Lloret J. (2019). An m-health application for cerebral stroke detection and monitoring using cloud services. Int. J. Inf. Manag..

[38] Mahajan R., Zafar S. (2021). DDos attacks impact on data transfer in IOT-MANET-based e-healthcare for tackling COVID-19. Data Analytics and Management.

[39] Habeeb R.A.A., Nasaruddin F., Gani A., Hashem I.A.T., Ahmed E., Imran M. (2019). Real-time big data processing for anomaly detection: A survey. Int. J. Inf. Manag..

[40] Shaik A., Borgaonkar R. New vulnerabilities in 5G networks. Proceedings of the Black Hat USA Conference.

[41] Gherbi C., Senouci O., Harbi Y., Medani K., Aliouat Z. (2023). A systematic literature review of machine learning applications in IoT. Int. J. Commun. Syst..

[42] Alzaidi M.S., Subbalakshmi C., Roshini T., Shukla P.K., Shukla S.K., Dutta P., Alhassan M. (2022). 5G-Telecommunication Allocation Network Using IoT Enabled Improved Machine Learning Technique. Wirel. Commun. Mob. Comput..

[43] Abranches M., Michel O., Keller E., Schmid S. Efficient Network Monitoring Applications in the Kernel with eBPF and XDP. Proceedings of the 2021 IEEE Conference on Network Function Virtualization and Software Defined Networks (NFV-SDN).

[44] Aziz M.F., Khan A.N., Shuja J., Khan I.A., Khan F.G., Khan A.u.R. (2022). A lightweight and compromise-resilient authentication scheme for IoTs. Trans. Emerg. Telecommun. Technol..

[45] Vishwakarma R., Jain A.K. (2020). A survey of DDoS attacking techniques and defence mechanisms in the IoT network. Telecommun. Syst..

[46] Chou E., Groves R. (2018). Distributed Denial of Service (DDoS).

[47] Ahmad R., Alsmadi I., Alhamdani W., Tawalbeh L.a. (2023). Zero-day attack detection: A systematic literature review. Artif. Intell. Rev..

[48] Prasad A., Chandra S. (2022). VMFCVD: An optimized framework to combat volumetric DDoS attacks using machine learning. Arab. J. Sci. Eng..

[49] David J., Thomas C. (2019). Efficient DDoS flood attack detection using dynamic thresholding on flow-based network traffic. Comput. Secur..

[50] Sreeram I., Vuppala V.P.K. (2019). HTTP flood attack detection in application layer using machine learning metrics and bio inspired bat algorithm. Appl. Comput. Inform..

[51] Liu X., Ren J., He H., Zhang B., Song C., Wang Y. (2021). A fast all-packets-based DDoS attack detection approach based on network graph and graph kernel. J. Netw. Comput. Appl..

[52] Yang L., Zhao H. DDoS attack identification and defense using SDN based on machine learning method. Proceedings of the 2018 15th International Symposium on Pervasive Systems, Algorithms and Networks (I-SPAN).

[53] Kirdan E., Raumer D., Emmerich P., Carle G. Building a traffic policer for ddos mitigation on top of commodity hardware. Proceedings of the 2018 International Symposium on Networks, Computers and Communications (ISNCC).

[54] Ramanathan S., Mirkovic J., Yu M., Zhang Y. SENSS against volumetric DDoS attacks. Proceedings of the 34th Annual Computer Security Applications Conference.

[55] Jaafar G.A., Abdullah S.M., Ismail S. (2019). Review of recent detection methods for HTTP DDoS attack. J. Comput. Netw. Commun..

[56] Smys S. (2019). DDOS attack detection in telecommunication network using machine learning. J. Ubiquitous Comput. Commun. Technol. UCCT.

[57] Kumar A. (2020). An Review on HTTP, TCP Flood, DDOS Attack in Cloud Environment & Their Solutions. Int. J. Sci. Res. Comput. Sci. Eng..

[58] Choe Y., Shin J.-S., Lee S., Kim J. eBPF/XDP based network traffic visualization and dos mitigation for intelligent service protection. Proceedings of the International Conference on Emerging Internetworking, Data & Web Technologies.

[59] Dimolianis M., Pavlidis A., Maglaris V. A multi-feature DDoS detection schema on P4 network hardware. Proceedings of the 2020 23rd Conference on Innovation in Clouds, Internet and Networks and Workshops (ICIN).

[60] Imthiyas M., Wani S., Abdulghafor R.A.A., Ibrahim A.A., Mohammad A.H. (2020). Ddos mitigation: A review of content delivery network and its ddos defence techniques. Int. J. Perceptive Cogn. Comput..

[61] Ghorbani H., Mohammadzadeh M.S., Ahmadzadegan M.H. DDoS Attacks on the IoT Network with the Emergence of 5G. Proceedings of the 2020 International Conference on Technology and Entrepreneurship-Virtual (ICTE-V).

[62] Peneti S., Hemalatha E. DDOS Attack Identification using Machine Learning Techniques. Proceedings of the 2021 International Conference on Computer Communication and Informatics (ICCCI).

[63] Awan M.J., Farooq U., Babar H.M.A., Yasin A., Nobanee H., Hussain M., Hakeem O., Zain A.M. (2021). Real-time DDoS attack detection system using big data approach. Sustainability.

[64] Thapa K., Duraipandian N. (2021). Malicious traffic classification using long short-term memory (LSTM) model. Wirel. Pers. Commun..

[65] Liu X., Ren J., He H., Wang Q., Song C. (2021). Low-rate DDoS attacks detection method using data compression and behavior divergence measurement. Comput. Secur..

[66] Thorat O., Parekh N., Mangrulkar R. (2021). TaxoDaCML: Taxonomy based Divide and Conquer using machine learning approach for DDoS attack classification. Int. J. Inf. Manag. Data Insights.

[67] Karpowicz M.P. (2021). Adaptive tuning of network traffic policing mechanisms for DDoS attack mitigation systems. Eur. J. Control.

[68] Zhan M., Li Y., Yang H., Yu G., Li B., Wang W. (2022). Coda: Runtime Detection of Application-Layer CPU-Exhaustion DoS Attacks in Containers. IEEE Trans. Serv. Comput..

[69] Szynkiewicz P. (2022). Signature-Based Detection of Botnet DDoS Attacks. Cybersecurity of Digital Service Chains.

[70] Makita T., Tu W., NSBU N.V. Faster OVS Datapath with XDP. Proceedings of the Netdev 0x14 Conference.

[71] Karlsson M., Töpel B. The path to DPDK speeds for AF XDP. Proceedings of the Linux Plumbers Conference.

[72] Alashhab A.A., Zahid M.S.M., Azim M.A., Daha M.Y., Isyaku B., Ali S. (2022). A Survey of Low Rate DDoS Detection Techniques Based on Machine Learning in Software-Defined Networks. Symmetry.

[73] Visky G., Vaarandi R. (2022). Performance and Applicability Analysis of Open-source Intrusion Detection Systems in Special-purpose Networks. Bachelor’s Thesis.

[74] Saleh A.J.M., Adnan N. Denial-of-Service (DoS) Threat Detection Using Supervised Machine Learning Algorithms on CICIDS2018 Dataset. Proceedings of the International Conference on Fourth Industrial Revolution and Beyond 2021.

[75] Abdulla N.N., Hasoun R.K. (2022). Review of Detection Denial of Service Attacks using Machine Learning through Ensemble Learning. Iraqi J. Comput. Inform..

[76] Oktivasari P., Zain A.R., Agustin M., Kurniawan A., Arbi Murad F., Fabian Anshor M. Analysis of Effectiveness of Iptables on Web Server from Slowloris Attack. Proceedings of the 2022 5th International Conference of Computer and Informatics Engineering (IC2IE).

[77] Florea R., Craus M. Modeling an Enterprise Environment for Testing Openstack Cloud Platform against Low-Rate DDoS Attacks. Proceedings of the 2022 26th International Conference on System Theory, Control and Computing (ICSTCC).

[78] Gaur V., Kumar R. (2022). Analysis of machine learning classifiers for early detection of DDoS attacks on IoT devices. Arab. J. Sci. Eng..

[79] Kattadige C., Choi K.N., Wijesinghe A., Nama A., Thilakarathna K., Seneviratne S., Jourjon G. (2021). Seta++: Real-time scalable encrypted traffic analytics in multi-gbps networks. IEEE Trans. Netw. Serv. Manag..

[80] Zhou X., Hu Y., Liang W., Ma J., Jin Q. (2020). Variational LSTM enhanced anomaly detection for industrial big data. IEEE Trans. Ind. Inform..

[81] Vieira M.A., Castanho M.S., Pacífico R.D., Santos E.R., Júnior E.P.C., Vieira L.F. (2020). Fast packet processing with ebpf and xdp: Concepts, code, challenges, and applications. ACM Comput. Surv. (CSUR).

[82] Tellis V.M., D’Souza D.J. Detecting anomalies in data stream using efficient techniques: A review. Proceedings of the 2018 International Conference on Control, Power, Communication and Computing Technologies (ICCPCCT).

[83] Scholz D., Raumer D., Emmerich P., Kurtz A., Lesiak K., Carle G. Performance implications of packet filtering with linux ebpf. Proceedings of the 2018 30th International Teletraffic Congress (ITC 30).

[84] Viegas E., Santin A., Bessani A., Neves N. (2019). BigFlow: Real-time and reliable anomaly-based intrusion detection for high-speed networks. Future Gener. Comput. Syst..

[85] Coulter R., Han Q.-L., Pan L., Zhang J., Xiang Y. (2019). Data-driven cyber security in perspective—Intelligent traffic analysis. IEEE Trans. Cybern..

[86] Miano S., Doriguzzi-Corin R., Risso F., Siracusa D., Sommese R. (2019). Introducing SmartNICs in server-based data plane processing: The DDoS mitigation use case. IEEE Access.

[87] Hu Q., Yu S.-Y., Asghar M.R. (2020). Analysing performance issues of open-source intrusion detection systems in high-speed networks. J. Inf. Secur. Appl..

[88] Cassagnes C., Trestioreanu L., Joly C., State R. The rise of eBPF for non-intrusive performance monitoring. Proceedings of the NOMS 2020-2020 IEEE/IFIP Network Operations and Management Symposium.

[89] Bu X., Sun Y.-E., Du Y., Wu X., Zhang B., Huang H. (2021). A novel spread estimation based abnormal flow detection in high-speed networks. Peer—Peer Netw. Appl..

[90] Han L., Guo Z., Huang X., Zeng X. (2021). A Multifunctional Full-Packet Capture and Network Measurement System Supporting Nanosecond Timestamp and Real-Time Analysis. IEEE Trans. Instrum. Meas..

[91] Farshin A., Barbette T., Roozbeh A., Maguire Jr G.Q., Kostić D. PacketMill: Toward per-Core 100-Gbps networking. Proceedings of the 26th ACM International Conference on Architectural Support for Programming Languages and Operating Systems.

[92] Salopek D. (2022). Hybrid Hardware/Software Datapath for Near Real-Time Reconfigurable High-Speed Packet Filtering. Ph.D. Thesis.

[93] Vittal S. (2022). HARNESS: High Availability supportive Self Reliant Network Slicing in 5G Networks. IEEE Trans. Netw. Serv. Manag..

[94] Bonola M., Belocchi G., Tulumello A., Brunella M.S., Siracusano G., Bianchi G., Bifulco R. Faster Software Packet Processing on {FPGA}{NICs} with {eBPF} Program Warping. Proceedings of the 2022 USENIX Annual Technical Conference (USENIX ATC 22).

[95] Wieren H. (2019). Signature-Based Ddos Attack Mitigation: Automated Generating Rules for Extended Berkeley Packet Filter and Express Data Path. Master’s Thesis.

[96] Li K., Lang B., Liu H., Chen S. (2022). SSL/TLS Encrypted Traffic Application Layer Protocol and Service Classification. CS IT Conf. Proc..

[97] Kumaigorodski A., Lutz C., Markl V. Fast CSV loading using GPUs and RDMA for in-memory data processing. Proceedings of the Datenbanksysteme für Business, Technologie und Web (BTW 2021).

[98] Zhang Z., Liu Z., Jiang Q., Chen J., An H. (2021). RDMA-based apache storm for high-performance stream data processing. Int. J. Parallel Program..

[99] Shapira A., Zolfi A., Demetrio L., Biggio B., Shabtai A. (2022). Denial-of-Service Attack on Object Detection Model Using Universal Adversarial Perturbation. arXiv.

[100] Ahalawat A., Babu K.S., Turuk A.K., Patel S. (2022). A low-rate DDoS detection and mitigation for SDN using Renyi Entropy with Packet Drop. J. Inf. Secur. Appl..

[101] Wang T., Yang X., Antichi G., Sivaraman A., Panda A. Isolation Mechanisms for High-Speed Packet-Processing Pipelines. Proceedings of the 19th USENIX Symposium on Networked Systems Design and Implementation (NSDI 22).

[102] Chiang J.-K., Lin Y.-C., Lin C.-W., Ting C.-S., Chiang Y.-Y., Kao Y.-H. (2022). Validation of snoring detection using a smartphone app. Sleep. Breath..

[103] Salva-Garcia P., Ricart-Sanchez R., Chirivella-Perez E., Wang Q., Alcaraz-Calero J.M. (2022). XDP-Based SmartNIC Hardware Performance Acceleration for Next-Generation Networks. J. Netw. Syst. Manag..

[104] Martínek T., Campanella M., FBK F.P., Hill J. (2022). White Paper: Timestamping and Clock Synchronisation in P4-Programmable Platforms.

[105] D’Alconzo A., Drago I., Morichetta A., Mellia M., Casas P. (2019). A survey on big data for network traffic monitoring and analysis. IEEE Trans. Netw. Serv. Manag..

[106] Melnick J. (2018). Top 10 most common types of cyber attacks. Netwrix Blog.

[107] Praseed A., Thilagam P.S. (2018). DDoS attacks at the application layer: Challenges and research perspectives for safeguarding web applications. IEEE Commun. Surv. Tutor..

[108] Ismail S., Hassen H.R., Just M., Zantout H. (2021). A review of amplification-based distributed denial of service attacks and their mitigation. Comput. Secur..

[109] Van Leeuwen B., Gao J., Yin H.K., Anthony B., Urias V. (2022). Networked-Based Cyber Analysis Using Deep Packet Inspection (DPI) for High-Speed Networks.

[110] Cerović D., Del Piccolo V., Amamou A., Haddadou K., Pujolle G. (2018). Fast packet processing: A survey. IEEE Commun. Surv. Tutor..

[111] Deepak A., Huang R., Mehra P. eBPF/XDP based firewall and packet filtering. Proceedings of the Linux Plumbers Conference.

[112] Li Z. HPSRouter: A high performance software router based on DPDK. Proceedings of the 2018 20th International Conference on Advanced Communication Technology (ICACT).

[113] Mohammadi R., Lal C., Conti M., Sharma L. (2022). Software defined network-based HTTP flooding attack defender. Comput. Electr. Eng..

[114] Cheema A., Tariq M., Hafiz A., Khan M.M., Ahmad F., Anwar M. (2022). Prevention Techniques against Distributed Denial of Service Attacks in Heterogeneous Networks: A Systematic Review. Secur. Commun. Netw..

[115] Deka R.K., Bhattacharyya D.K., Kalita J.K. (2019). Active learning to detect DDoS attack using ranked features. Comput. Commun..

[116] Sharafaldin I., Lashkari A.H., Hakak S., Ghorbani A.A. Developing realistic distributed denial of service (DDoS) attack dataset and taxonomy. Proceedings of the 2019 International Carnahan Conference on Security Technology (ICCST).

[117] Boeder C., Januchowski T. (2022). Zero-day DDoS Attack Detection. arXiv.

[118] Black S., Kim Y. An Overview on Detection and Prevention of Application Layer DDoS Attacks. Proceedings of the 2022 IEEE 12th Annual Computing and Communication Workshop and Conference (CCWC).

[119] Sadqi Y., Maleh Y. (2022). A systematic review and taxonomy of web applications threats. Inf. Secur. J. A Glob. Perspect..

[120] Liu Z., Namkung H., Nikolaidis G., Lee J., Kim C., Jin X., Braverman V., Yu M., Sekar V. Jaqen: A High-Performance Switch-Native Approach for Detecting and Mitigating Volumetric DDoS Attacks with Programmable Switches. Proceedings of the 30th USENIX Security Symposium (USENIX Security 21).

